# Supplementary material for: EcoCountHelper: an R package and analytical pipeline for the analysis of ecological count data using GLMMs, and a case study of bats in Grand Teton National Park
Source: PeerJ. 2022 Dec 14;10:e14509. doi: 10.7717/peerj.14509 (PMC9758971; doi:10.7717/peerj.14509)
Supplement: Supplemental Information 1 — Supplementary information for “EcoCountHelper: an R package and analytical pipeline for the analysis of ecological count data using GLMMs, and a case study of bats in Grant Teton National Park”. [file peerj-10-14509-s001.docx]

**MATERIALS & METHODS**

*Anthropogenic Roost Suitability*

To determine buildings that were suitable as bat day roosts, we examined all National Park Service-owned buildings within ~1 kilometer of our study sites. Building descriptions included construction materials (e.g., log walls, standing seam roof), any openings found in the building’s exterior surfaces that would provide a sheltered area for bats to roost, and any evidence of bat inhabitation. In addition, we also photographed all building features. We then used written descriptions and photographs to code buildings as either suitable or not for bat inhabitation. To parameterize the effect of potential roost-building presence near monitoring sites, we created a spatially-weighted potential roost-building density index using the formula

$$\sum_{i =1}^{n} \frac{1}{x_{i}}$$

where *n* represents the number of roost buildings classified as being suitable for bat inhabitation within one kilometer of a given monitoring site, and *x* represents the distance from a building to a given monitoring site. Because bat home ranges are poorly understood, this cutoff distance of one kilometer was somewhat arbitrary. Data on North American bat home range size is limited, and the literature that does exist suggests that home range sizes vary substantially based on variables including location, sex, reproductive status, and home-range estimation method. *Myotis lucifugus*, for example, has been estimated to have home ranges from 17.6 hectares (Henry et al., 2002) to 2,739 hectares (Bergeson, 2012). Our cutoff distance of one kilometer would result in a ~314 hectare circular area surrounding each monitoring site. While this may be an over- or under-approximation of home range size for bats in Grand Teton, the spatial-weighting we implemented offers some protection from imperfections in the cutoff distance.

*Land Cover*

To allow us to assess the impact of habitat characteristics on bat activity, we quantified the proportions of forested and developed areas comprising a 50-meter buffer surrounding each site. A 50 meter buffer was chosen for potential drivers of local activity to reflect the approximate range that our monitoring units were capable of recording low frequency bat echolocation (Agranat, 2014) rather than the 1 kilometer buffer that was used to quantify roost availability provided by artificial structures within commuting distance of our sites. While National Landcover Database (NLCD) data is frequently used for this purpose (Agranat, 2014), we thought it would be inappropriate to use such a low resolution dataset for our spatial scale of analysis. In lieu of using NLCD data, we manually defined and classified polygons of forested and developed areas using high-resolution Google satellite imagery and QGIS 3.10. Using the resultant shapefile, we calculated the proportion of a 50-meter buffer comprised of forested and developed areas.

*Distance to Water*

To assess the importance of proximity to water sources, we calculated the distance to the nearest persistent stream, river, lake, or pond. Shapefiles from the National Hydrography Dataset (NHD), namely the Waterbody and Flowline shapefiles, were used as spatially referenced water features. Flowlines (streams and rivers) were truncated to Fcodes 46006 and 55800 to only include persistent and substantial flowing water features. We then calculated the minimum distance from each site to any portion of a water feature.

*Elevation*

To assess the importance of elevation, we used ⅓ arc-second USGS DEMs (Gesch et al., 2002) to sample the elevation of each monitoring site.

*Moon Illumination*

To control for the effect of lunar illumination on bat activity (Saldaña-Vázquez & Munguía-Rosas, 2013), we used the `lunar` (Lazaridis, 2014) package in R (R Core Team, 2020) to calculate the proportion of the moon illuminated on each night of data collection.

*Artificial Light Sources*

Following site visits to describe buildings, we re-visited sites at night to document artificial light sources. For each light within ~500 meters of a monitoring site, we qualitatively described the color (e.g., orange, yellow-white, blue-white) and brightness of the light, and the coordinates of the light in decimal degrees. Because some types of lights look similar but have different underlying spectra (e.g., tungsten filament and compact fluorescent bulbs), we did not attempt to extrapolate beyond the visual appearance of the light. We scored the brightness of each light on a 1-5 scale by comparing artificial light sources to two reference lights (an REI collapsible lamp and a Maglite Mini LED flashlight with the reflector and lens removed). The dimmer REI collapsible lamp represented a two on our scale, and the brighter Maglite represented a four on our scale. All artificial light sources were documented, including light sources that were likely operated by a switch (e.g., porch lights) under the assumption that a sample of lights on any given night would be representative of the lightscape in an area during the summer. Because we were unable to collect radiometric data for light sources included in this analysis, we collapsed the color of artificial lights to a binary categorical variable based on apparent color temperature (cool or warm) to ensure that our assessment of light color was accurate and as objective as possible. We then calculated the proportion of lights classified as having a cool color temperature within a 50 meter buffer surrounding each site. Because the number of lights surrounding a site and the sum of brightness scores were highly collinear, we were forced to include only one of those predictors in our analyses. We chose to include the sum of brightness scores within a 50-meter radius of each monitoring site because we thought it better reflects the magnitude of artificial light’s impact on an area compared to a simple count of light fixtures.

*Bat Call Sequence Processing*

We analyzed bat echolocation sequences using the SonoBat 4.3.0 software suite and checked files for frequency-modulated (FM) sweeps using the SonoBat Data Wizard which moves files without FM sweeps (indicative of the absence of bat echolocation) to subdirectories that are not included in the proceeding steps of data preparation. We used Sonobat’s Western Wyoming call library to truncate possible species to those found in our study area and set the following parameters for SonoBat to handle and identify call sequences: 10 kHz high-pass filter; acceptable call quality = 0.80; sequence decision threshold = 0.90; maximum number of calls to consider per file = 32. Following data processing with SonoBat, we aggregated output files containing file names and species identifications by site-night and species using R. After aggregating for each species, we appended spatial and temporal predictor data to each site-night of data processed by SonoBat.

**REFERENCES**

Agranat I. 2014. Detecting bats with ultrasonic microphones: understanding the effects of microphone variance and placement on detection rates. *Unpublished white paper. Wildlife Acoustics, Maynard, MA*:209–256.

Bergeson SM. 2012. Examining the suitability of the little brown bat (Myotis lucifugus) as a surrogate for the endangered Indiana bat (M. sodalis).

Gesch D, Oimoen M, Greenlee S, Nelson C, Steuck M, Tyler D. 2002. The national elevation dataset. *Photogrammetric engineering and remote sensing* 68:5–32.

Henry M, Thomas DW, Vaudry R, Carrier M. 2002. Foraging distances and home range of pregnant and lactating little brown bats (Myotis lucifugus). *Journal of Mammalogy* 83:767–774.

Lazaridis E. 2014. *lunar: Lunar Phase & Distance, Seasons and Other Environmental Factors*.

R Core Team. 2020. *R: A Language and Environment for Statistical Computing*. Vienna, Austria: R Foundation for Statistical Computing.

Saldaña-Vázquez RA, Munguía-Rosas MA. 2013. Lunar phobia in bats and its ecological correlates: A meta-analysis. *Mammalian Biology* 78:216–219. DOI: 10.1016/j.mambio.2012.08.004.
