# Supplementary material for: EcoCountHelper: an R package and analytical pipeline for the analysis of ecological count data using GLMMs, and a case study of bats in Grand Teton National Park
Source: PeerJ. 2022 Dec 14;10:e14509. doi: 10.7717/peerj.14509 (PMC9758971; doi:10.7717/peerj.14509)
Supplement: Supplemental Information 2 [file peerj-10-14509-s002.html]

Analyzing Wildlife Count Data using EcoCountHelper


# Analyzing Wildlife Count Data using EcoCountHelper

#### May 10, 2021

- Package Purpose
- Understanding GLMMs and Reading Materials
- Installing and Citing EcoCountHelper
  - Installing
  - Citing
- Requisite Data Preparation & Model Construction
  - Basic Data and Model Structure for glmmTMB Models
  - Modeling with glmmTMB
    - Conditional Models
    - Error Distributions and Link Functions
    - Zero-inflated Models
    - Writing Functions for Modeling Multiple Groups
- Identifying Best Fit Models
  - Saving and Loading Models
- Checking Goodness-of-Fit
- Plotting Effects
- Examining “Real-World” Effects
  - Single Model and Predictor
  - Multiple Models and Predictors
- Formatting “RealEffects” Tables
- Long-form Analysis
- References

## Package Purpose

`EcoCountHelper` is a R package designed to make the meaningful analysis of wildlife count data through generalized linear mixed-models (GLMMs) more accessible to land and wildlife managers with limited programmatic experience. GLMMs can be a powerful and flexible tool for analyzing hierarchical data (e.g., repeated counts of bat call sequences at multiple sites), but the lack of standardization surrounding these analyses paired with the requisite level of programmatic competence can make them daunting to approach for many biologists and ecologists. The `EcoCountHelper` package provides a structured framework for approaching GLMM analyses that simplifies the coding process necessary to conduct such analyses.

## Understanding GLMMs and Reading Materials

Put very simply, GLMMs are a linear modeling technique that allow researchers to assess the effect of both numeric and categorical predictors on a response variable while allowing one to account for non-independence of data (e.g., repeated measures of a response variable that come from the same location) through random effects, and to specify the error distribution a model should use (e.g., Poisson, Gaussian, negative binomial). It is of paramount importance that anyone who uses this package understands the fundamentals of GLMMs. There are no programmatic safeguards that prevent poorly-constructed models from being used to draw scientific conclusions, and thus it is imperative that anyone who intends to use `EcoCountHelper` first familiarizes themselves with the basics of GLMM analysis. Rather than rehashing points made in expertly-written overviews of GLMMs, we highly recommend (at a minimum) fully reading the following cited texts before going forward with any GLMM analyses (Bolker et al., 2009; Harrison et al., 2018).

Furthermore, understanding the fundamentals of zero-inflation and GLMMs incorporating zero-inflated models will be beneficial for properly modeling many wildlife count datasets. A dataset is zero-inflated when there are substantially more zeros present than a specified model structure and error distribution predict. To account for zeros that are not predicted by the conditional model, zero-inflated models can be used to model the process by which additional zeros arise. More information on zero-inflation, zero-inflated negative binomial (ZINB) models, and zero-inflated Poisson (ZIP) models can be found in the following references (Martin et al., 2005; Brooks et al., 2017b).

Finally, it is important to understand how to use the R package `glmmTMB`, the package through which users of `EcoCountHelper` must construct their models, before using `EcoCountHelper`. Information regarding the use and syntax of `glmmTMB` can be found in the following cited documents (Bolker, 2016; Brooks et al., 2017b; Brooks et al., 2017a).

## Installing and Citing EcoCountHelper

### Installing

While obvious, it is important to have an appropriate version of R installed on your machine. The earliest version of R that this package has been tested with is 3.6.3, and thus we recommend updating your R install if you are using an earlier version of R.

The simplest way to install `EcoCountHelper` is to use the `install_github` function from the `devtools` package. Before proceeding with this the installation of EcoCountHelper, you must first ensure that you have the software “Rtools” installed (available through this link), then ensure `devtools` is installed. To install and load `devtools` then install `EcoCountHelper`, use the code below:

```
install.packages("devtools")
require(devtools)
install_github(repo = "huntercole25/EcoCountHelper", build_vignettes = T)
```

### Citing

After installing `EcoCountHelper`, to see citation information for the package use the line of code below:

```
citation("EcoCountHelper")
```

## Requisite Data Preparation & Model Construction

### Basic Data and Model Structure for glmmTMB Models

Much like the equations many people were exposed to in elementary algebra classes, glmmTMB **conditional models** have two sides; the left side contains the response variable, and the right side contains predictors (explanatory variables). A conditional model is the formula that specifies the predictors for which probability distributions (and consequentially coefficients) will be developed in relation to the response variable. Throughout this example we will be analyzing a dataset pertaining to bat activity throughout Grand Teton National Park. In this case, bat activity (call sequences recorded per site-night) will be the response variable on the left side of the conditional model formula, and explanatory variables such as elevation and distance to water will be on the right side of the conditional model formula. A coefficient describing the linear relationship between the predictor and the response variable will be calculated for each predictor on the right side of the formula.

In order to model the data described above, a data frame or data table will need to be prepared that has a vector (column) for the response variable, as well as a vector for each predictor that will be used in the conditional model. Wildlife count data often contain multiple response variables (e.g., ultrasonic passive acoustic monitoring data may contain call sequence counts for multiple bat species, avian point counts may contain counts for multiple bird species). To demonstrate one method of incorporating multiple response variables into a single data frame, let’s take a look at the *wide-form* Grand Teton bat data:

```
library(EcoCountHelper)
data("BatDataWide", package = "EcoCountHelper")
head(BatDataWide)
```

```
##    Site SampleDate Epfu Laci Lano Myev Mylu Myvo Myyu EastingUsAea
## 1: 12PM 2016-07-08    0    6   41    2    7    0    0     -1164933
## 2: 12PM 2016-07-09    0   23    1    0   26    1    0     -1164933
## 3: 12PM 2016-07-10    0    1    0    0    1    0    0     -1164933
## 4: 12PM 2016-07-11    0    1    0    0    0    0    0     -1164933
## 5: 12PM 2016-07-12    1    9    0    0    0    0    0     -1164933
## 6: 12PM 2016-07-13    0    1    0    0    2    0    0     -1164933
##    NorthingUsAea Year Yday   YdayScale   MoonPct   MoonScale ManualForestPct
## 1:      805272.1 2016  190 -0.10044831 0.1440887 -0.48706986               0
## 2:      805272.1 2016  191 -0.08337973 0.2262721 -0.37151734               0
## 3:      805272.1 2016  192 -0.06631115 0.3208006 -0.23860716               0
## 4:      805272.1 2016  193 -0.04924257 0.4234110 -0.09433357               0
## 5:      805272.1 2016  194 -0.03217400 0.5294755  0.05479669               0
## 6:      805272.1 2016  195 -0.01510542 0.6342107  0.20205785               0
##    ForestScale ManualDevelPct DevelScale WaterDist WaterScale     Elev
## 1:  -0.4486677              0 -0.2200783         0 -0.2542275 2081.289
## 2:  -0.4486677              0 -0.2200783         0 -0.2542275 2081.289
## 3:  -0.4486677              0 -0.2200783         0 -0.2542275 2081.289
## 4:  -0.4486677              0 -0.2200783         0 -0.2542275 2081.289
## 5:  -0.4486677              0 -0.2200783         0 -0.2542275 2081.289
## 6:  -0.4486677              0 -0.2200783         0 -0.2542275 2081.289
##     ElevScale BrightCount BrightScale PropCool  CoolScale StrWeight   StrScale
## 1: 0.08695775           0   -0.220249        0 -0.1576591         0 -0.3538291
## 2: 0.08695775           0   -0.220249        0 -0.1576591         0 -0.3538291
## 3: 0.08695775           0   -0.220249        0 -0.1576591         0 -0.3538291
## 4: 0.08695775           0   -0.220249        0 -0.1576591         0 -0.3538291
## 5: 0.08695775           0   -0.220249        0 -0.1576591         0 -0.3538291
## 6: 0.08695775           0   -0.220249        0 -0.1576591         0 -0.3538291
```

For more information regarding what each vector in the data above represents, please run the following line of code: `?BatDataWide`. For information regarding data collection methods, please see Cole et al., 2021.

In this case, the response variables (named with the four-letter codes for each species; Epfu, Laci, Lano, Myev, Mylu, Myvo and Myyu) each have their own vector in the data table, and each site-night occupies a single row. When data containing multiple response variables is formatted in this way, it is in wide-form. Another acceptable data format is *long-form*, with each combination of the unit of replication (in this case site-nights) and response variable occupying their own rows, a vector specifying the response variable (in this case, species) that a row corresponds to, and a vector detailing the value of the response variable (in this case, count). Here is a long-form version of the data shown above:

```
data("BatDataLong", package = "EcoCountHelper")
head(BatDataLong)
```

```
##    Site SampleDate Species Count EastingUsAea NorthingUsAea Year Yday
## 1: 12PM 2016-07-08    Epfu     0     -1164933      805272.1 2016  190
## 2: 12PM 2016-07-08    Laci     6     -1164933      805272.1 2016  190
## 3: 12PM 2016-07-08    Lano    41     -1164933      805272.1 2016  190
## 4: 12PM 2016-07-08    Myev     2     -1164933      805272.1 2016  190
## 5: 12PM 2016-07-08    Mylu     7     -1164933      805272.1 2016  190
## 6: 12PM 2016-07-08    Myvo     0     -1164933      805272.1 2016  190
##     YdayScale   MoonPct  MoonScale ManualForestPct ForestScale ManualDevelPct
## 1: -0.1004483 0.1440887 -0.4870699               0  -0.4486677              0
## 2: -0.1004483 0.1440887 -0.4870699               0  -0.4486677              0
## 3: -0.1004483 0.1440887 -0.4870699               0  -0.4486677              0
## 4: -0.1004483 0.1440887 -0.4870699               0  -0.4486677              0
## 5: -0.1004483 0.1440887 -0.4870699               0  -0.4486677              0
## 6: -0.1004483 0.1440887 -0.4870699               0  -0.4486677              0
##    DevelScale WaterDist WaterScale     Elev  ElevScale BrightCount BrightScale
## 1: -0.2200783         0 -0.2542275 2081.289 0.08695775           0   -0.220249
## 2: -0.2200783         0 -0.2542275 2081.289 0.08695775           0   -0.220249
## 3: -0.2200783         0 -0.2542275 2081.289 0.08695775           0   -0.220249
## 4: -0.2200783         0 -0.2542275 2081.289 0.08695775           0   -0.220249
## 5: -0.2200783         0 -0.2542275 2081.289 0.08695775           0   -0.220249
## 6: -0.2200783         0 -0.2542275 2081.289 0.08695775           0   -0.220249
##    PropCool  CoolScale StrWeight   StrScale
## 1:        0 -0.1576591         0 -0.3538291
## 2:        0 -0.1576591         0 -0.3538291
## 3:        0 -0.1576591         0 -0.3538291
## 4:        0 -0.1576591         0 -0.3538291
## 5:        0 -0.1576591         0 -0.3538291
## 6:        0 -0.1576591         0 -0.3538291
```

Both long- and wide- form data can be used to build GLMMs using `glmmTMB`, and both formats are also compatible with `EcoCountHelper`. Note that in both data formats, each row provides a response value as well as the values for all predictors on a given site-night. With an understanding of long- and wide-form data established, we will be using the example wide-form data, “BatDataWide,” for the majority of the remainder of this example. After the entire process associated with `EcoCountHelper` has been addressed using wide-form data, we will provide an explanation of the small changes to the workflow required to use long-form data with this package.

Notice that all numeric predictors have a second associated vector that is named with the suffix “Scale.” All of these predictors have been standardized which puts all data on the same scale. When vectors with different units of measurement are standardized, their parameter estimates are more directly comparable. Model convergence is often more easily attained when numeric predictor vectors are scaled. A good resource for information on standardizing data can be found here.

Data are frequently standardized by subtracting the mean of a vector from all data in that vector and dividing all values by the vector’s standard deviation. This type of standardizing can be accomplished with base R’s `scale` function. The data shown above are scaled by subtracting the mean from all values then dividing by *two* standard deviations. This can be done using EcoCountHelper’s `scale2` function. By dividing all data in a vector by two standard deviations of that vector, parameter estimates for all standardized variables are directly comparable to those of categorical (factor) variables with two levels (Gelman, 2008). In the data used in this example, “Year” has two levels (2016 and 2017). By standardizing all numeric predictors by subtracting the mean for each vector and dividing by two standard deviations, we are able to directly compare the magnitude and surrounding confidence of the parameter estimates for all continuous variables in our models as well as the binary categorical variable “Year.” All numeric variables have already been standardized using `scale2` in the previously loaded data, but an example of how we calculated the standardized “MoonScale” vector using the “MoonPct” and `scale2` function is shown below.

```
# data.frame syntax
BatDataWide$MoonScale <- scale2(BatDataWide$MoonPct)

# data.table syntax
BatDataWide[,MoonScale := scale2(MoonPct)]
```

Once you’ve identified variables that may influence your response variable, all predictor and response variable data are prepared appropriately in either long- or wide-form and all vectors are formatted appropriately, you are ready to begin building your GLMMs.

### Modeling with glmmTMB

#### Conditional Models

`glmmTMB` uses Wilkinson notation for specifying formulas which follows the description outlined at the beginning of the section above. We’ll use both data formats shown above (long- and wide-form) to demonstrate the model building process. First, we’ll decide on a base conditional model formula to be used for all groups in the analysis. In this example, a group is a bat species, and we will be using the same conditional model parameterization for each group in the analysis. Our literature review and knowledge of our study system suggested that our models should include a metric of artificial roost habitat availability (StrScale), metrics of artificial light presence and characteristics (BrightScale and CoolScale), ordinal date (YdayScale), elevation (ElevScale), distance to the nearest water source (WaterScale), metrics of developed and forested land presence surrounding monitoring sites (DevelScale and ForestScale), and lunar illumination (MoonScale) as fixed-effect predictors of species-specific bat activity levels. Because we gathered repeated measures of bat activity from multiple sites and across multiple years, we also included site as a random effect (specified as (1|Site)) and year as a fixed effect. If possible, year should be included as a random intercept, but with only two levels of a year variable, we opted not to include year as a random intercept term (Gelman & Hill, 2006). Below is the base conditional model we used for all species in this example, with “SpCount” representing the response vector (call-sequences recorded per night) for a given bat species in the example data:

```
SpCount ~  StrScale + CoolScale + BrightScale + Year + YdayScale + ElevScale +
                     WaterScale + DevelScale + ForestScale + MoonScale + (1|Site)
```

#### Error Distributions and Link Functions

The data fitting process for GLMMs uses iteratively reweighted least squares (IRLS) to calculate maximum likelihood estimates for model covariates. Put more simply, GLMMs implement IRLS to produce the coefficients associated with each predictor specified in the conditional model. The IRLS process, and consequently the specification of models using `glmmTMB`, requires users to specify a mean-variance relationship (error distribution) for the response variable in order to calculate the weights of individual residuals in estimate calculations. Specifying the correct error distribution is important because different error distributions will produce different residual values, and thus different residual weights, and ultimately different parameter estimates.

While choosing the correct error distribution for your models may seem intimidating, it can actually be a relatively straightforward process. While more computationally intensive than other similar methods, we suggest generating models implementing multiple plausible error distributions, then corroborating qualitative assessments of mean-variance plots with AIC values to determine which error-distribution family should be used for the final stages of analysis. For count data (non-negative integers), three plausible (and commonly used) error distributions are Poisson (`poisson()` from the `stats` package), negative-binomial with a linear parameterization (`nbinom1()` from the `glmmTMB` package), and negative-binomial with a quadratic parameterization (`nbinom2()` from the `glmmTMB` package). We suggest that users create a model for each combination of group (in this case species) and the error-distributions listed above when preparing models for use with `EcoCountHelper`. Using the conditional model shown above, and using the “family” argument of the `glmmTMB` function to specify error-distributions, the set of models for a species of bat may look like the code below:

```
# Poisson
glmmTMB(SpCount ~  StrScale + CoolScale + BrightScale + Year + YdayScale + ElevScale +
                     WaterScale + DevelScale + ForestScale + MoonScale + (1|Site),
        family = poisson(link = "log"))

# Negative-binomial with linear parameterization
glmmTMB(SpCount ~  StrScale + CoolScale + BrightScale + Year + YdayScale + ElevScale +
                     WaterScale + DevelScale + ForestScale + MoonScale + (1|Site),
        family = nbinom1(link = "log"))

# Negative-binomial with quadratic parameterization
glmmTMB(SpCount ~  StrScale + CoolScale + BrightScale + Year + YdayScale + ElevScale +
                     WaterScale + DevelScale + ForestScale + MoonScale + (1|Site),
        family = nbinom2(link = "log"))
```

Notice that the link argument in each error-distribution function is “log.” A link in this context is a function specifying the transformation of the response variable’s *mean* so that a linear relationship between the expected values of the response variable and the predictors can be established. Because the link function maintains the probability distribution of a response variable by transforming its mean (expected value) given a particular condition (set of predictor values), a properly specified link function allows for the preservation of both the process that generated the data and a linear relationship between predictors and the transformed response. The natural logarithm link specified above is appropriate for count data because log-link function is only compatible with non-negative numbers (e.g., count data), and it puts the transformed mean of responses on a continuous scale which allows for linear modeling of the data. Unless you have a compelling reason to do so and are very familiar with GLMMs, we suggest leaving all link functions for the error-distributions listed above as “log.”

#### Zero-inflated Models

The error distribution family specification described above provides a mean-variance relationship for GLMM estimate calculations, but occasionally count data exhibit overdispersion due to there being more data points with a value of zero than the specified conditional model and error distribution may expect. Conceptually, this may be caused by separate processes generating zero and non-zero values. Consider the example data: perhaps early in our sampling periods there are an excess of zero counts because bats have not yet migrated back to their summer ranges, and perhaps some sites simply aren’t suitable for a given bat species to inhabit them for reasoons unparameterized by our conditional model. There may be an excess of zeros early in our sampling periods or at certain sites, but when there is detectable bat activity it can be described by a Poisson or negative-binomial distribution. If your data contains a high number of zeros, and consequently your data exhibits overdispersion relative to Poisson or negative-binomial distributions, it is advantageous to specify a zero-inflated model *in addition to* your conditional model. It is important to understand that a zero-inflated model is not a different type of model than your standard GLMM, but rather an addendum to your specified error-distribution that allows it to expect a high number of zeros given certain conditions (associated with a separate zero-generating process) as well as a distribution of non-zero values according to the “family” argument specification.

While checking for zero-inflation after identifying an appropriate error distribution is possible, it can be unnecessarily time consuming and manually intensive. Instead, we recommend creating two models for each error distribution specified in the previous step and assessing goodness of fit for all error distributions as well as zero-inflated models simultaneously. The final set of candidate models for a group (in this case, bat species), assuming excess zeros are generated by a process involving ordinal date and sampling site, will look like the code below:

```
# Non-zero-inflated
glmmTMB(SpCount ~  StrScale + CoolScale + BrightScale + Year + YdayScale + ElevScale +
                     WaterScale + DevelScale + ForestScale + MoonScale + (1|Site),
        family = poisson(link = "log"))

glmmTMB(SpCount ~  StrScale + CoolScale + BrightScale + Year + YdayScale + ElevScale +
                     WaterScale + DevelScale + ForestScale + MoonScale + (1|Site),
        family = nbinom1(link = "log"))

glmmTMB(SpCount ~  StrScale + CoolScale + BrightScale + Year + YdayScale + ElevScale +
                     WaterScale + DevelScale + ForestScale + MoonScale + (1|Site),
        family = nbinom2(link = "log"))

# Zero-inflated
glmmTMB(SpCount ~  StrScale + CoolScale + BrightScale + Year + YdayScale + ElevScale +
                     WaterScale + DevelScale + ForestScale + MoonScale + (1|Site),
        family = poisson(link = "log"), ziformula = ~YdayScale + Site)

glmmTMB(SpCount ~  StrScale + CoolScale + BrightScale + Year + YdayScale + ElevScale +
                     WaterScale + DevelScale + ForestScale + MoonScale + (1|Site),
        family = nbinom1(link = "log"), ziformula = ~YdayScale + Site)

glmmTMB(SpCount ~  StrScale + CoolScale + BrightScale + Year + YdayScale + ElevScale +
                     WaterScale + DevelScale + ForestScale + MoonScale + (1|Site),
        family = nbinom2(link = "log"), ziformula = ~YdayScale + Site)
```

#### Writing Functions for Modeling Multiple Groups

In total, we advise generating six models for each response group included in the analysis. Depending on the number of response groups you plan to include in your analysis, writing the code for each model individually can become a cumbersome task. In this example, we have seven species included in our analysis for a total of 42 models to generate. Additionally, when running code for each model individually, keeping track of which models converge is tedious. Rather than writing the code for each model and supervising the modeling process, we often write functions that iteratively run the six models described above (three common error distributions for count data - Poisson, negative-binomial with a linear parameterization, and negative-binomial with a quadratic parameterization - each run both with and without a zero-inflated formula) for each response group, dynamically name each model generated, and classify models as having converged or not. Below is the code necessary to iteratively generate six models for each of the seven bat species we are examining the activity of in this example.

```
SpeciesList <- c("Epfu", "Laci", "Lano", "Myev", "Mylu", "Myvo", "Myyu")

WideDataModeler <- function(Data, Species){
  BatData <- get(deparse(substitute(Data)))
  
  SpNb1 <- tryCatch({glmmTMB(BatData[[Species]] ~ StrScale + CoolScale + BrightScale + Year +
                               YdayScale + ElevScale + WaterScale + DevelScale + ForestScale +
                               MoonScale + (1|Site),
                   data = BatData, family = nbinom1(link = "log"))},
                   error = function(cond){return(NA)},
                   warning = function(cond){return(NA)})
  
  assign(paste0(Species, "_Nb1"), SpNb1, pos = .GlobalEnv)
  
  SpNb2 <- tryCatch({glmmTMB(BatData[[Species]] ~ StrScale + CoolScale + BrightScale + Year +
                               YdayScale + ElevScale + WaterScale + DevelScale + ForestScale +
                               MoonScale + (1|Site),
                   data = BatData, family = nbinom2(link = "log"))},
                   error = function(cond){return(NA)},
                   warning = function(cond){return(NA)})
  
  assign(paste0(Species, "_Nb2"), SpNb2, pos = .GlobalEnv)
  
  SpPoi <- tryCatch({glmmTMB(BatData[[Species]] ~ StrScale + CoolScale + BrightScale + Year +
                               YdayScale + ElevScale + WaterScale + DevelScale + ForestScale +
                               MoonScale + (1|Site),
                   data = BatData, family = poisson(link = "log"))},
                   error = function(cond){return(NA)},
                   warning = function(cond){return(NA)})
  
  assign(paste0(Species, "_Poi"), SpPoi, pos = .GlobalEnv)
  
  SpZiNb1 <- tryCatch({glmmTMB(BatData[[Species]] ~ StrScale + CoolScale + BrightScale + Year +
                               YdayScale + ElevScale + WaterScale + DevelScale + ForestScale +
                               MoonScale + (1|Site),
                   data = BatData, family = nbinom1(link = "log"), ziformula = ~YdayScale + Site)},
                   error = function(cond){return(NA)},
                   warning = function(cond){return(NA)})
  
  assign(paste0(Species, "_ZiNb1"), SpZiNb1, pos = .GlobalEnv)

  SpZiNb2 <- tryCatch({glmmTMB(BatData[[Species]] ~ StrScale + CoolScale + BrightScale + Year +
                               YdayScale + ElevScale + WaterScale + DevelScale + ForestScale +
                               MoonScale + (1|Site),
                     data = BatData, family = nbinom2(link = "log"), ziformula = ~YdayScale + Site)},
                     error = function(cond){return(NA)},
                     warning = function(cond){return(NA)})
  
  assign(paste0(Species, "_ZiNb2"), SpZiNb2, pos = .GlobalEnv)
  
  SpZiPoi <- tryCatch({glmmTMB(BatData[[Species]] ~ StrScale + CoolScale + BrightScale + Year +
                               YdayScale + ElevScale + WaterScale + DevelScale + ForestScale +
                               MoonScale + (1|Site),
                     data = BatData, family = poisson(link = "log"), ziformula = ~YdayScale + Site)},
                     error = function(cond){return(NA)},
                     warning = function(cond){return(NA)})
  
  assign(paste0(Species, "_ZiPoi"), SpZiPoi, pos = .GlobalEnv)
}
```

Some of the functions (including the `function()` function) may be unfamiliar to you, so we’ll break down the code above. The first line, `SpeciesList <- c("Epfu", "Laci", "Lano", "Myev", "Mylu", "Myvo", "Myyu")` specifies the response vectors that we will be generating models for. Each of these response vectors contains count data describing the number of call sequences recorded for the bat species specified by the four letter code comprising the vector name.

To explain the user-defined function, we will build from the most basic components toward the end product.

```
SpeciesList <- c("Epfu", "Laci", "Lano", "Myev", "Mylu", "Myvo", "Myyu")

WideDataModeler <- function(Data, Species){
  
}
```

In the code chunk directly above we are creating a function that will be assigned to the global environment as “WideDataModeler” that will have two arguments: Data and Species. Anything in the curly brackets in the following code chunks will be executed when the function is called.

```
SpeciesList <- c("Epfu", "Laci", "Lano", "Myev", "Mylu", "Myvo", "Myyu")

WideDataModeler <- function(Data, Species){
  BatData <- get(deparse(substitute(Data)))
  
    SpNb1 <- glmmTMB(BatData[[Species]] ~ StrScale + CoolScale + BrightScale + Year +
                               YdayScale + ElevScale + WaterScale + DevelScale + ForestScale +
                               MoonScale + (1|Site),
                   data = BatData, family = nbinom1(link = "log"))
    
    SpNb2 <- glmmTMB(BatData[[Species]] ~ StrScale + CoolScale + BrightScale + Year +
                               YdayScale + ElevScale + WaterScale + DevelScale + ForestScale +
                               MoonScale + (1|Site),
                   data = BatData, family = nbinom2(link = "log"))
  
  SpPoi <- glmmTMB(BatData[[Species]] ~ StrScale + CoolScale + BrightScale + Year +
                               YdayScale + ElevScale + WaterScale + DevelScale + ForestScale +
                               MoonScale + (1|Site),
                   data = BatData, family = poisson(link = "log"))
  
  SpZiNb1 <- glmmTMB(BatData[[Species]] ~ StrScale + CoolScale + BrightScale + Year +
                               YdayScale + ElevScale + WaterScale + DevelScale + ForestScale +
                               MoonScale + (1|Site),
                   data = BatData, family = nbinom1(link = "log"), ziformula = ~YdayScale + Site)

  SpZiNb2 <- glmmTMB(BatData[[Species]] ~ StrScale + CoolScale + BrightScale + Year +
                               YdayScale + ElevScale + WaterScale + DevelScale + ForestScale +
                               MoonScale + (1|Site),
                     data = BatData, family = nbinom2(link = "log"), ziformula = ~YdayScale + Site)

  SpZiPoi <- glmmTMB(BatData[[Species]] ~ StrScale + CoolScale + BrightScale + Year +
                               YdayScale + ElevScale + WaterScale + DevelScale + ForestScale +
                               MoonScale + (1|Site),
                     data = BatData, family = poisson(link = "log"), ziformula = ~YdayScale + Site)
}
```

Immediately above we’ve added a line to read in the data that will be specified in the “Data” argument as well as calls to create glmmTMB objects for each of the six models we discussed in the zero-inflation section. Notice that the specified response variable in all models is “BatData[[Species]].” When we use this function to iteratively model count data for our response groups listed in the “SpeciesList” object, using “BatData[[Species]]” as the response variable will allow our function to dynamically call the vectors named with the character strings listed in the “SpeciesList” object. Also note that each model is assigned with a name that describes the error distribution used in the model, and the presence of a zero-inflated formula if applicable (denoted by the “Zi” preceeding the error distribution abbreviations in the last three models). Any objects created within the function do not leave the function’s environment (i.e., function-created objects do not enter the global environment) without an explicit call to do so (as is shown immediately below), and any function-generated objects are removed from the function’s environment upon completion of the function’s execution.

```
SpeciesList <- c("Epfu", "Laci", "Lano", "Myev", "Mylu", "Myvo", "Myyu")

WideDataModeler <- function(Data, Species){
  BatData <- get(deparse(substitute(Data)))
  
  SpNb1 <- glmmTMB(BatData[[Species]] ~ StrScale + CoolScale + BrightScale + Year +
                               YdayScale + ElevScale + WaterScale + DevelScale + ForestScale +
                               MoonScale + (1|Site),
                   data = BatData, family = nbinom1(link = "log"))
  
  assign(paste0(Species, "_Nb1"), SpNb1, pos = .GlobalEnv)
  
  SpNb2 <- glmmTMB(BatData[[Species]] ~ StrScale + CoolScale + BrightScale + Year +
                               YdayScale + ElevScale + WaterScale + DevelScale + ForestScale +
                               MoonScale + (1|Site),
                   data = BatData, family = nbinom2(link = "log"))
  
  assign(paste0(Species, "_Nb2"), SpNb2, pos = .GlobalEnv)
  
  SpPoi <- glmmTMB(BatData[[Species]] ~ StrScale + CoolScale + BrightScale + Year +
                               YdayScale + ElevScale + WaterScale + DevelScale + ForestScale +
                               MoonScale + (1|Site),
                   data = BatData, family = poisson(link = "log"))
  
  assign(paste0(Species, "_Poi"), SpPoi, pos = .GlobalEnv)
  
  SpZiNb1 <- glmmTMB(BatData[[Species]] ~ StrScale + CoolScale + BrightScale + Year +
                               YdayScale + ElevScale + WaterScale + DevelScale + ForestScale +
                               MoonScale + (1|Site),
                   data = BatData, family = nbinom1(link = "log"), ziformula = ~YdayScale + Site)
  
  assign(paste0(Species, "_ZiNb1"), SpZiNb1, pos = .GlobalEnv)

  SpZiNb2 <- glmmTMB(BatData[[Species]] ~ StrScale + CoolScale + BrightScale + Year +
                               YdayScale + ElevScale + WaterScale + DevelScale + ForestScale +
                               MoonScale + (1|Site),
                     data = BatData, family = nbinom2(link = "log"), ziformula = ~YdayScale + Site)
  
  assign(paste0(Species, "_ZiNb2"), SpZiNb2, pos = .GlobalEnv)
  
  SpZiPoi <- glmmTMB(BatData[[Species]] ~ StrScale + CoolScale + BrightScale + Year +
                               YdayScale + ElevScale + WaterScale + DevelScale + ForestScale +
                               MoonScale + (1|Site),
                     data = BatData, family = poisson(link = "log"), ziformula = ~YdayScale + Site)
  
  assign(paste0(Species, "_ZiPoi"), SpZiPoi, pos = .GlobalEnv)
```

The `assign()` calls above dynamically name the models that were assigned to the function’s environment through use of the `paste0()` function, and assign them to the global environment so they will persist beyond the execution of the function. Developing an object naming protocol to use throughout the workflow we will be using throughout this example is imperative. Giving objects descriptive names with a consistent structure not only provides information regarding the object, but also allows one to retrieve that object from the global environment using regular expressions. Multiple `EcoCountHelper` functions use regular expressions to retrieve objects from the global environment with minimal user input, so using names with patterns that are recognizable to regular expressions is integral to the use of this package. Perhaps the easiest means of denoting response group membership in an object name with regular-expression-friendly structuring is to begin a name with the group membership code (in this case, the four letter bat species code) followed by an underscore, then a description of the nature of the object. The model names generated by the `assign()` calls above demonstrate this naming scheme.

The final step to completing the function initially outlined is to add the `tryCatch()` calls.

```
SpeciesList <- c("Epfu", "Laci", "Lano", "Myev", "Mylu", "Myvo", "Myyu")

WideDataModeler <- function(Data, Species){
  BatData <- get(deparse(substitute(Data)))
  
  SpNb1 <- tryCatch({glmmTMB(BatData[[Species]] ~ StrScale + CoolScale + BrightScale + Year +
                               YdayScale + ElevScale + WaterScale + DevelScale + ForestScale +
                               MoonScale + (1|Site),
                   data = BatData, family = nbinom1(link = "log"))},
                   error = function(cond){return(NA)},
                   warning = function(cond){return(NA)})
  
  assign(paste0(Species, "_Nb1"), SpNb1, pos = .GlobalEnv)
  
  SpNb2 <- tryCatch({glmmTMB(BatData[[Species]] ~ StrScale + CoolScale + BrightScale + Year +
                               YdayScale + ElevScale + WaterScale + DevelScale + ForestScale +
                               MoonScale + (1|Site),
                   data = BatData, family = nbinom2(link = "log"))},
                   error = function(cond){return(NA)},
                   warning = function(cond){return(NA)})
  
  assign(paste0(Species, "_Nb2"), SpNb2, pos = .GlobalEnv)
  
  SpPoi <- tryCatch({glmmTMB(BatData[[Species]] ~ StrScale + CoolScale + BrightScale + Year +
                               YdayScale + ElevScale + WaterScale + DevelScale + ForestScale +
                               MoonScale + (1|Site),
                   data = BatData, family = poisson(link = "log"))},
                   error = function(cond){return(NA)},
                   warning = function(cond){return(NA)})
  
  assign(paste0(Species, "_Poi"), SpPoi, pos = .GlobalEnv)
  
  SpZiNb1 <- tryCatch({glmmTMB(BatData[[Species]] ~ StrScale + CoolScale + BrightScale + Year +
                               YdayScale + ElevScale + WaterScale + DevelScale + ForestScale +
                               MoonScale + (1|Site),
                   data = BatData, family = nbinom1(link = "log"), ziformula = ~YdayScale + Site)},
                   error = function(cond){return(NA)},
                   warning = function(cond){return(NA)})
  
  assign(paste0(Species, "_ZiNb1"), SpZiNb1, pos = .GlobalEnv)

  SpZiNb2 <- tryCatch({glmmTMB(BatData[[Species]] ~ StrScale + CoolScale + BrightScale + Year +
                               YdayScale + ElevScale + WaterScale + DevelScale + ForestScale +
                               MoonScale + (1|Site),
                     data = BatData, family = nbinom2(link = "log"), ziformula = ~YdayScale + Site)},
                     error = function(cond){return(NA)},
                     warning = function(cond){return(NA)})
  
  assign(paste0(Species, "_ZiNb2"), SpZiNb2, pos = .GlobalEnv)
  
  SpZiPoi <- tryCatch({glmmTMB(BatData[[Species]] ~ StrScale + CoolScale + BrightScale + Year +
                               YdayScale + ElevScale + WaterScale + DevelScale + ForestScale +
                               MoonScale + (1|Site),
                     data = BatData, family = poisson(link = "log"), ziformula = ~YdayScale + Site)},
                     error = function(cond){return(NA)},
                     warning = function(cond){return(NA)})
  
  assign(paste0(Species, "_ZiPoi"), SpZiPoi, pos = .GlobalEnv)
}
```

The addition of the `tryCatch()` calls above force models that did not converge or exhibitted singular convergence (which causes an error or warning) to a value of NA rather than a glmmTMB class object. By allowing only models that did not have model convergence issues to maintain their glmmTMB class, we can use the `Filter()` function later to list the names of all viable models in the global environment for further analysis.

Running the code in the last code chunk will assign the function we assigned in the global environment as “WideDataModeler.” With our modeling function defined and our data prepared, we can finally use `lapply()` to iteratively generate the six models defined in our function for each of the response groups we are examining.

```
lapply(SpeciesList, WideDataModeler, Data = BatDataWide)
```

## Identifying Best Fit Models

After defining a function to generate multiple models for a given bat species and using `lapply()` to iteratively generate those models for each of our response groups, we can now begin identifying the model that best fits the data for each species. There are two methods that we use to identify best-fitting models for each response group: AIC value comparison and mean-variance plot examination. The order that these steps are performed in does not matter, as information from both methods will be corroborated to identify the best-fitting model for each species.

In this example we’ll begin with mean-variance plots. These plots are not particularly difficult to construct manually, however we have built a set of functions (the “DistFit” family of functions) that facilitate the process. For wide data, the function for this process is `DistFitWide()`. These functions aggregate data by one or more factor variables (in the case of this example, we’ll use the “Year” and “Site” vectors to aggregate data) for each response group that is supplied in the character vector provided to the “GroupList” argument. “GroupList” values must specify the response vectors as named in the data provided to the “Data” argument.

```
DistFitWide(Splitters = c("Year", "Site"), Data = BatDataWide,
            GroupList = SpeciesList)
```

Rather than showing the plot for each response group under investigation (seven in total), we will only show the plot for *Eptesicus fuscus* for the time being. Because each plot produced is named using the response group name (“Epfu” in the case of *E. fuscus*) followed by “DistPlot,” we can use the code below to call the *E. fuscus* mean-variance plot.

```
EpfuDistPlot
```

The points plotted in this graph show individual mean and variance data for each site-year combination, and the lines running through the data show the different error distributions discussed above. By examining each plot and how well each error distribution fits the mean-variance relationship exhibited by the points, we can ascertain the best error distribution to use for each response group.

`EcoCountHelper` also has a function that produces tabulated AIC values for each response group, as well as a table showing the best fitting model (as defined by AIC) for each response group. Users specify the names of the response groups under investigation (which should also be in the model names as mentioned above) using the “Groups” argument, and the desired name of the table listing the top model for each response group using the “TopModOut” argument.

```
ModelCompare(Groups = SpeciesList, TopModOutName = BestFitModels)
```

The `ModelCompare()` function names each species’ AIC table starting with the response group name followed by “AIC,” so to call the AIC table for *E. fuscus*, we would use the first line of the code below. We can also call the table containing the top model for each response group under investigation using the name provided to the “TopModOut” argument in the `ModelCompare()` call made above (in this case, the table is named “BestFitModels”).

```
EpfuAIC
```

```
##         Model      Aic Subj
## 1: Epfu_ZiPoi 4012.652 Epfu
## 2:   Epfu_Poi 4443.152 Epfu
## 3:   Epfu_Nb2 2961.711 Epfu
## 4:   Epfu_Nb1 3001.302 Epfu
```

```
BestFitModels
```

```
##    Subj TopModel
## 1: Myyu Myyu_Nb2
## 2: Myvo Myvo_Nb2
## 3: Mylu Mylu_Nb2
## 4: Myev Myev_Nb2
## 5: Lano Lano_Nb2
## 6: Laci Laci_Nb2
## 7: Epfu Epfu_Nb2
```

With both a mean-variance plot and AIC values for each response variable in our analysis, we can now corroborate each of these to arrive at the best error-distribution to use for each response group, as well as whether or not we should use a zero-inflated model for each response group. In the case of mean-variance plots and AIC values suggesting different error distributions, we suggest using the mean-variance plot to inform your decision. Additionally, because the mean-variance plots do not speak to the presence of zero-inflation, we use solely the AIC values for each response group to determine whether to use a zero-inflated model for each response group.

If the “top model table” generated by the `ModelCompare()` function does not accurately reflect the models you have concluded best fit your data, you can easily modify individual values. For instance, if we concluded that using a negative-binomial error distribution with a linear parameterization best fit the *E. fuscus* data, we could use the line of code below to reflect that decision in our table of top models.

```
BestFitModels[BestFitModels$Subj == "Epfu"][["TopModel"]] <- "Epfu_Nb1" #data.frame syntax

BestFitModels[Subj == "Epfu", TopModel := "Epfu_Nb1"] #data.table syntax
```

### Saving and Loading Models

Because running models can take a substantial amount of time, it’s worth saving at least the models that you will use for the remainder of your analysis. Models can be saved and named dynamically with a simple loop like the one below:

```
SaveDir <- "Z:/Models" # Specifying a pre-existing directory to save to

for(i in unique(BestFitModels$TopModel)){ # Iterates through the models in the "BestFitModels" table
  saveRDS(get(i, pos = .GlobalEnv), file = paste0(SaveDir, "/", i, ".rds")) # Saves the model with the name it has in your global environment as the file name
}
```

We could also save all glmmTMB-class objects in the environment to .rds files using the code below:

```
SaveDir <- "Z:/Models" # Specifying a pre-existing directory to save to

# Creates a character vector of object names with class "glmmTMB"
Mods <- Filter(function(x) inherits(get(x), "glmmTMB"), ls(pos = .GlobalEnv))

# Iteratively saves models to .rds files in the directory specified above
for(i in Mods){
  tmp <- get(i)
  saveRDS(tmp, file = paste0(SaveDir, "/", i, ".rds"))
}
```

If you close your R session and would like to reload all of the models you have saved to a directory, you can use the code below after specifying the directory you saved .rds files to in the first line of the chunk:

```
LoadDir <- "Z:/Models" # Specifying a pre-existing directory to load from

for(i in list.files(LoadDir, full.names = T, pattern = "\\.rds")){
  TmpMod <- readRDS(i)
  assign(regmatches(basename(i), regexpr("^[[:alnum:]]+_[[:alnum:]]+",
                                         basename(i))), TmpMod)
}
```

## Checking Goodness-of-Fit

While the models we chose as the best fits for the data in the step above were, in fact, the best of the choices provided, they still may not be adequate to use for inference due to poor overall fit. `DHARMa` is a package that allows users to run diagnostic tests and create diagnostic plots for the simulated residuals of models. `EcoCountHelper` contains the “ResidPlot” family of functions which creates useful diagnostic plots to assess goodness-of-fit. For wide data, we can use the `ResidPlotWide()` function as is shown below. The “Data” argument allows users to specify the data frame or data table from which the models were generated, and the “ModNames” argument allows users to specify a vector of models to be examined. The output will be three plots for each model provided to the “ModNames” argument that will be saved as a single object in the global environment named with the model name followed by “SimResidPlot.”

```
ResidPlotWide(Data = BatDataWide, ModNames = BestFitModels$TopModel)
```

To call the *E. fuscus* residual diagnostic plots, we can use the code below:

```
Epfu_Nb2SimResidPlot
```

Without providing too much detail, the QQ-plot (the left-most plot) should show a line of points that are relatively straight, and that fall close to the red line running diagonally across the plot. The dispersion plot (the middle plot) should show a histogram with a peak close to the vertical red line shown in the plot. The residual outlier distribution (the right-most plot) should show a relatively flat histogram with few or no bars filled with red. For more information on what these plots mean, please see this DHARMa vignette. Additionally, it is important to note that, because these plots are based on simulations, no two function calls will result in identical plots, although the results should be very similar and lead to the same conclusion regarding goodness-of-fit.

## Plotting Effects

We’ve built our candidate models, chosen the ones that best fit the data, and assessed goodness-of-fit. We can now assume the results of our models are valid, and proceed with the model interpretation process. `EcoCountHelper` has multiple functions for model interpretation. One of the first steps in the data interpretation process is to examine the relative importance of predictors. This step of the process can be accomplished with the `EffectsPlotter()` function which plots the parameter estimates for each predictor along with specified confidence intervals surrounding those estimates. Much the same as other functions in this package, we supply a list of models to the “TopMods” argument to specify which models should be used to create effects plots. We also need to specify the labels for each estimate plotted. This can be done in the same order as the labels produced by `row.names(summary(x)$coeff$cond)`, where x is the name of a model. While it may produce non-intuitive labels for people unfamiliar with the analysis, we can also supply `row.names(summary(x)$coeff$cond)` to the “ParamLabs” argument. The function defaults to displaying 80% and 90% confidence intervals.

```
EffectsPlotter(TopMods = BestFitModels$TopModel,
               ParamLabs = rownames(summary(Epfu_Nb2)$coeff$cond))
```

All of the plots generated by the model call above named plots by appending “EffectsPlot” to the end of each model name supplied to the “TopMods” argument. We can view the effects plot for *E. fuscus* using the line of code below.

```
Epfu_Nb2EffectsPlot
```

This plot can be easier to interpret with more descriptive labels for each predictor. We can change the parameter labels by supplying a character vector of labels to the “ParamLabs” argument, with the labels in the same order that the predictors are returned by `row.names(summary(x)$coeff$cond)`.

```
EffectsPlotter(TopMods = BestFitModels$TopModel,
               ParamLabs = c("Intercept (Site)", "Structure Index",
                             "Prop. Cool", "Brightness Index", "Year (16-17)",
                             "Ordinal Date", "Elevation", "Dist. to Water", 
                             "Prop. Developed", "Prop. Forest", "Moon Illum."))
```

```
Epfu_Nb2EffectsPlot
```

Now is a good time to see if a part of the model results used to generate the plot above makes sense - the intercept value. It is very important check to make sure your intercept value makes sense because your intercept should be around your mean value for the response: if all continuous variables are scaled, then the intercept is the mean value when all continuous variables are at their mean (0 for each in this case) and all categorical variables are set to their respective lowest value in alphabetical or numeric order (2016 in the case of the “Year” predictor in these models). Because of the log link used in these models, this intercept value is the natural logarithm of the predicted response variable value with all numeric predictors set at their mean value, and all categorical predictors set at their first value in ascending alphabetical or numeric value. To check whether the intercept value in the plot above (-0.1682902) makes sense, we can use the base R’s “exp()” function to exponentiate out intercept value, and compare that exponentiated value to the mean and/or median of the response variable. Given that `exp(-0.1682902)` yields a value of 0.845, the median of the “Epfu” vector is 1, and the mean of the “Epfu” vector is 3.183, we can assume that this intercept value is quite reasonable. While some derivation of the intercept from metrics of central tendency for the response variable is acceptable, an intercept value of 5 (148 when exponentiated) would have been cause for concern.

In this plot, we can also see that there appears to be a strong and positive relationship between *E. fuscus* activity and ordinal date, year, and our structure index. Because we built these models with standardized numeric data, however, we cannot directly interpret the “real world” effect sizes of any of these parameters from the effects plots. For models with log links, in order to assess the factor by which a given change in a predictor changes the predicted response variable value, we must exponentiate Euler’s number by the coefficient of interest, then exponentiate that number by a specified unit of change. While not particularly complicated, carrying these calculations out manually can be arduous. To relieve users of some of the programmatic burden associated with transforming model coefficients into meaningful metrics of change, the `EcoCountHelper` package has a “RealEffects” family of functions that calculate the factor and percent by which a response variable changes for a given change in one or more predictor variables.

## Examining “Real-World” Effects

### Single Model and Predictor

To determine the predicted effect of a specified change in a single predictor on a response variable, we can use the `RealEffectsText()` function, which calculates the predicted change in a response variable as a percentage or a factor (depending on the magnitude of change). To use this function with an unscaled predictor, we must specify the model we would like to use for predicting the impact of a change in a predictor using the “Model” argument, the predictor of interest using the “Predictor” argument, and the unstandardized quantity that the predictor should be changed by. For standardized predictors, we also must include how many deviations the data were divided by during the standardizing process using the “ScaleSds” argument, and the vector of unstandardized data from which the standardized was calculated using the “PredVect” argument. The code chunk below predicts the change in nightly *E. fuscus* call sequences for a 20% increase in forest cover at the monitoring site. Note that we supply a “ScaleSds” value of 2 because we used `scale2()` to standardize the raw ManualForestPct vector.

```
RealEffectText(Model = Epfu_Nb2, Predictor = "ForestScale",
               UnitChange = 0.2, ScaleSds = 2,
               PredVect = BatDataWide$ManualForestPct)
```

```
## [1] "The response variable increases 19.08% (+/- 53.66%) for every 0.2 unit increase in the predictor."
```

Note that the confidence interval values default to 95% confidence intervals. You can specify alternative confidence interval values using the “ConfInt” argument. Also note that the specified change in the predictor should only be expressed as a proportion/percent value if the unstandardized data for that predictor were collected as a proportion/percent value. Had we used the standardized “WaterScale” and unstandardized “WaterDist” as the vectors for the predictor of interest, we would have supplied the “UnitChange” argument with a value in meters since the unstandardized “WaterDist” vector expresses values in meters.

### Multiple Models and Predictors

With models for seven bat species and nine numeric predictors for each model, using `RealEffectText()` 63 times to obtain predicted changes in each species’ activity for a change in each predictor would be tedious, and would require the manual tabulation of results. To allow users to easily create a table with predicted changes in responses for multiple response groups and changes in multiple predictors, we created `RealEffectTabWide()` and `RealEffectTabLong()` which tabulate changes in response variables for all combinations derived from a vector of models and a vector of common predictors. The arguments for the “RealEffectTab” functions are nearly the same as those associated with `RealEffectText()`, but most arguments accept vectors rather than single values to calculate predicted response changes for multiple response groups with a single function call.

Because we are working with wide-form data in this example, we will use `RealEffectTabWide()`. Rather than specifying a single model, the “Models” argument accepts a character vector of models to be used for predicting response variable changes. As we did for all of the function calls above that required a vector of model names, we can use the vector of best fit models generated by our previous use of `ModelCompare()`. Use of the “Predictors” argument is identical in both `RealEffectText()` and `RealEffectTabWide()`, requiring a character vector listing predictors to change the value of for response change calculations. The “UnitChanges” argument accepts a numeric vector of values to change each predictor by in response variable change calculations. The vector supplied to the “UnitChanges” argument should be the same length as the “Predictors” vector, with one value of change for each predictor in the same order as predictors were listed in the “Predictors” vector. Similarly, if any of the predictors supplied to the “Predictors” argument are standardized, the “ScaleSds” must be specified as a numeric vector listing the number of standard deviations data were divided by during the standardization process. If predictors contain a mixture of standardized and unstandardized predictors, “ScaleSds” values associated with unstandardized predictors should be “NA.” The use of the “PredVects” argument in `RealEffectTabWide()` is the same as the use of the “PredVect” argument in `RealEffectText()`, but rather than supplying a single unstandardized vector to associate with a standardized predictor, the “PredVects” argument accepts a character vector of unstandardized vector names. If the “Predictors” argument contains both standardized and unstandardized predictors, “PredVects” values should be approached in the same way as “ScaleSds” in that values associated with unstandardized predictors should be assigned a value of “NA.” Finally, we need to provide the unquoted name of the data frame or data table to which all of the vectors names supplied above belong using the “Data” argument. To visualize all of the vectors supplied to the arguments above and ensure each predictor has the correct value change, standard deviations by which is was standardized and associated unstandardized vector, we can create a data frame or data table containing the vectors for all `RealEffectTabWide()` arguments.

```
RealEffectsArgs <- data.table(Predictors = c("MoonScale", "ForestScale", "DevelScale",
                                          "WaterScale", "ElevScale", "YdayScale",
                                          "BrightScale", "CoolScale", "StrScale"),
                              UnitChanges = c(0.2,  0.2, 0.2, 250, 50, 30, 5, 0.2, 0.05),
                              ScaleSds = rep(2, 9),
                              PredVects = c("MoonPct", "ManualForestPct", "ManualDevelPct",
                                "WaterDist", "Elev", "Yday", "BrightCount", "PropCool",
                                "StrWeight"))

RealEffectsArgs
```

```
##     Predictors UnitChanges ScaleSds       PredVects
## 1:   MoonScale        0.20        2         MoonPct
## 2: ForestScale        0.20        2 ManualForestPct
## 3:  DevelScale        0.20        2  ManualDevelPct
## 4:  WaterScale      250.00        2       WaterDist
## 5:   ElevScale       50.00        2            Elev
## 6:   YdayScale       30.00        2            Yday
## 7: BrightScale        5.00        2     BrightCount
## 8:   CoolScale        0.20        2        PropCool
## 9:    StrScale        0.05        2       StrWeight
```

We can now use the vectors we created above as argument values in the proceeding `RealEffectTabWide()` call.

```
RealEffectsTabExample <- RealEffectTabWide(Models = BestFitModels$TopMod,
                                           Predictors = RealEffectsArgs$Predictors,
                                           UnitChanges = RealEffectsArgs$UnitChanges,
                                           ScaleSds = RealEffectsArgs$ScaleSds,
                                           PredVects = RealEffectsArgs$PredVects,
                                           Data = BatDataWide)

RealEffectsTabExample
```

```
##        Model   Predictor UnitChange DeltaPct LowerConf UpperConf Pval
##  1: Myyu_Nb2   MoonScale       0.20     6.34    -10.63     23.30 0.44
##  2: Myyu_Nb2 ForestScale       0.20     0.65    -71.18     72.49 0.98
##  3: Myyu_Nb2  DevelScale       0.20    15.09   -178.78    208.96 0.80
##  4: Myyu_Nb2  WaterScale     250.00   -22.99    -63.25     17.27 0.13
##  5: Myyu_Nb2   ElevScale      50.00   -52.19  -1033.85    929.48 0.54
##  6: Myyu_Nb2   YdayScale      30.00    56.04      1.51    110.58 0.05
##  7: Myyu_Nb2 BrightScale       5.00   -16.15   -271.20    238.89 0.79
##  8: Myyu_Nb2   CoolScale       0.20    28.74    -36.16     93.64 0.32
##  9: Myyu_Nb2    StrScale       0.05     3.24    -54.63     61.12 0.89
## 10: Myvo_Nb2   MoonScale       0.20    -2.09     -8.29      4.11 0.49
## 11: Myvo_Nb2 ForestScale       0.20    89.11     43.80    134.41 0.00
## 12: Myvo_Nb2  DevelScale       0.20     4.78   -111.13    120.70 0.91
## 13: Myvo_Nb2  WaterScale     250.00   -12.45    -34.91     10.00 0.20
## 14: Myvo_Nb2   ElevScale      50.00   -29.53   -437.02    377.95 0.67
## 15: Myvo_Nb2   YdayScale      30.00     0.50    -19.38     20.39 0.96
## 16: Myvo_Nb2 BrightScale       5.00     4.82   -156.97    166.61 0.92
## 17: Myvo_Nb2   CoolScale       0.20    -9.83    -59.02     39.36 0.61
## 18: Myvo_Nb2    StrScale       0.05    63.43     27.83     99.03 0.00
## 19: Mylu_Nb2   MoonScale       0.20     9.57      4.58     14.56 0.00
## 20: Mylu_Nb2 ForestScale       0.20    57.22     -1.45    115.89 0.05
## 21: Mylu_Nb2  DevelScale       0.20    23.65   -110.64    157.95 0.63
## 22: Mylu_Nb2  WaterScale     250.00   -22.56    -50.62      5.50 0.04
## 23: Mylu_Nb2   ElevScale      50.00   -61.84   -701.87    578.19 0.35
## 24: Mylu_Nb2   YdayScale      30.00    85.22     66.43    104.01 0.00
## 25: Mylu_Nb2 BrightScale       5.00    48.34   -157.43    254.11 0.49
## 26: Mylu_Nb2   CoolScale       0.20    28.41    -36.70     93.53 0.33
## 27: Mylu_Nb2    StrScale       0.05    -0.63    -47.22     45.95 0.97
## 28: Myev_Nb2   MoonScale       0.20    -1.02     -6.02      3.98 0.68
## 29: Myev_Nb2 ForestScale       0.20     9.88    -30.35     50.10 0.59
## 30: Myev_Nb2  DevelScale       0.20   -33.34   -126.69     60.01 0.23
## 31: Myev_Nb2  WaterScale     250.00   -13.66    -33.73      6.41 0.12
## 32: Myev_Nb2   ElevScale      50.00   -53.73   -390.18    282.72 0.31
## 33: Myev_Nb2   YdayScale      30.00   101.53     84.69    118.37 0.00
## 34: Myev_Nb2 BrightScale       5.00    39.28    -93.62    172.18 0.44
## 35: Myev_Nb2   CoolScale       0.20     8.25    -36.73     53.23 0.68
## 36: Myev_Nb2    StrScale       0.05   -19.97    -54.43     14.48 0.14
## 37: Lano_Nb2   MoonScale       0.20    -6.02    -12.16      0.11 0.04
## 38: Lano_Nb2 ForestScale       0.20     2.29    -57.63     62.21 0.92
## 39: Lano_Nb2  DevelScale       0.20     8.04   -133.60    149.67 0.86
## 40: Lano_Nb2  WaterScale     250.00    -7.95    -36.15     20.25 0.51
## 41: Lano_Nb2   ElevScale      50.00   -45.01   -690.08    600.07 0.56
## 42: Lano_Nb2   YdayScale      30.00   135.27    114.49    156.06 0.00
## 43: Lano_Nb2 BrightScale       5.00    13.04   -202.89    228.98 0.83
## 44: Lano_Nb2   CoolScale       0.20   -17.24    -83.68     49.20 0.47
## 45: Lano_Nb2    StrScale       0.05    34.97    -12.21     82.15 0.13
## 46: Laci_Nb2   MoonScale       0.20     1.59     -4.59      7.77 0.61
## 47: Laci_Nb2 ForestScale       0.20   -17.35    -59.64     24.94 0.29
## 48: Laci_Nb2  DevelScale       0.20   -12.11   -111.07     86.84 0.71
## 49: Laci_Nb2  WaterScale     250.00    -9.54    -29.96     10.88 0.29
## 50: Laci_Nb2   ElevScale      50.00   146.15   -198.61    490.90 0.24
## 51: Laci_Nb2   YdayScale      30.00   523.11    498.79    547.43 0.00
## 52: Laci_Nb2 BrightScale       5.00    29.22   -111.16    169.60 0.57
## 53: Laci_Nb2   CoolScale       0.20   -19.74    -65.73     26.24 0.25
## 54: Laci_Nb2    StrScale       0.05    24.46     -9.73     58.64 0.14
## 55: Epfu_Nb2   MoonScale       0.20    -4.39    -11.95      3.17 0.23
## 56: Epfu_Nb2 ForestScale       0.20    19.08    -34.58     72.74 0.43
## 57: Epfu_Nb2  DevelScale       0.20   -29.44   -176.37    117.49 0.45
## 58: Epfu_Nb2  WaterScale     250.00   -12.54    -38.62     13.53 0.26
## 59: Epfu_Nb2   ElevScale      50.00    39.45   -482.12    561.02 0.72
## 60: Epfu_Nb2   YdayScale      30.00    69.54     43.76     95.32 0.00
## 61: Epfu_Nb2 BrightScale       5.00    50.02   -158.81    258.85 0.48
## 62: Epfu_Nb2   CoolScale       0.20   -28.30    -86.68     30.08 0.16
## 63: Epfu_Nb2    StrScale       0.05    82.29     40.79    123.79 0.00
##        Model   Predictor UnitChange DeltaPct LowerConf UpperConf Pval
```

In the table above, the vectors (from left to right) list the model, predictor, how much the predictor was changed, the percent by which the response is predicted to change for the given change in the predictor, the lower and upper confidence interval values for the predicted change in the response, and the p-value of the parameter estimate for the given model and predictor. The values in the table above are in long-form, which is convenient for indexing to view the results for all predictors within a single model or a single predictor across all models. We can try each of those indexing methods below.

```
# Viewing all predicted response changes for E. fuscus
RealEffectsTabExample[Model == "Epfu_Nb2",]
```

```
##       Model   Predictor UnitChange DeltaPct LowerConf UpperConf Pval
## 1: Epfu_Nb2   MoonScale       0.20    -4.39    -11.95      3.17 0.23
## 2: Epfu_Nb2 ForestScale       0.20    19.08    -34.58     72.74 0.43
## 3: Epfu_Nb2  DevelScale       0.20   -29.44   -176.37    117.49 0.45
## 4: Epfu_Nb2  WaterScale     250.00   -12.54    -38.62     13.53 0.26
## 5: Epfu_Nb2   ElevScale      50.00    39.45   -482.12    561.02 0.72
## 6: Epfu_Nb2   YdayScale      30.00    69.54     43.76     95.32 0.00
## 7: Epfu_Nb2 BrightScale       5.00    50.02   -158.81    258.85 0.48
## 8: Epfu_Nb2   CoolScale       0.20   -28.30    -86.68     30.08 0.16
## 9: Epfu_Nb2    StrScale       0.05    82.29     40.79    123.79 0.00
```

```
#Viewing all predicted response changes for a 20% increase in forest cover
RealEffectsTabExample[Predictor == "ForestScale",]
```

```
##       Model   Predictor UnitChange DeltaPct LowerConf UpperConf Pval
## 1: Myyu_Nb2 ForestScale        0.2     0.65    -71.18     72.49 0.98
## 2: Myvo_Nb2 ForestScale        0.2    89.11     43.80    134.41 0.00
## 3: Mylu_Nb2 ForestScale        0.2    57.22     -1.45    115.89 0.05
## 4: Myev_Nb2 ForestScale        0.2     9.88    -30.35     50.10 0.59
## 5: Lano_Nb2 ForestScale        0.2     2.29    -57.63     62.21 0.92
## 6: Laci_Nb2 ForestScale        0.2   -17.35    -59.64     24.94 0.29
## 7: Epfu_Nb2 ForestScale        0.2    19.08    -34.58     72.74 0.43
```

## Formatting “RealEffects” Tables

While viewing the output from `RealEffectTabWide()` calls in long-form can be convenient for quickly indexing values, wide-form results may be easier to integrate into a report in an easy-to-read and aesthetically pleasing way. The package `tidyr` should have been installed to your local library when `EcoCountHelper` was installed. We can use the `pivot_wider()` function from `tidyr` to make our results wide. We can also concatenate some of the vectors (“DeltaPct,” “LowerConf” and “UpperConf”) and create a vector of species to use for identifying response group membership instead of model names - both of which may make our table easier to read outside of R.

```
library(tidyr)

# Creates vector of species names from first four characters of model name
RealEffectsTabExample[,Species := substr(Model, 1, 4)] 

# Concatenates response change, and upper and lower confidence intervals into a single
# vector with symbols for added clarity
RealEffectsTabExample[DeltaPct >= 0, PctConf := paste0("+", DeltaPct,
                                        "% (", LowerConf,
                                        "% / ", UpperConf, "%)")]

RealEffectsTabExample[DeltaPct < 0, PctConf := paste0(DeltaPct,
                                       "% (", LowerConf, "% / ",
                                       UpperConf, "%)")]

# Creates wide-form results table with species as columns
RealEfWide <- as.data.table(pivot_wider(RealEffectsTabExample, id_cols = c(Predictor, UnitChange),
                                        names_from = Species, values_from = PctConf))

RealEfWide
```

```
##      Predictor UnitChange                          Myyu
## 1:   MoonScale       0.20      +6.34% (-10.63% / 23.3%)
## 2: ForestScale       0.20     +0.65% (-71.18% / 72.49%)
## 3:  DevelScale       0.20  +15.09% (-178.78% / 208.96%)
## 4:  WaterScale     250.00    -22.99% (-63.25% / 17.27%)
## 5:   ElevScale      50.00 -52.19% (-1033.85% / 929.48%)
## 6:   YdayScale      30.00     +56.04% (1.51% / 110.58%)
## 7: BrightScale       5.00   -16.15% (-271.2% / 238.89%)
## 8:   CoolScale       0.20    +28.74% (-36.16% / 93.64%)
## 9:    StrScale       0.05     +3.24% (-54.63% / 61.12%)
##                            Myvo                         Mylu
## 1:      -2.09% (-8.29% / 4.11%)      +9.57% (4.58% / 14.56%)
## 2:    +89.11% (43.8% / 134.41%)   +57.22% (-1.45% / 115.89%)
## 3:   +4.78% (-111.13% / 120.7%) +23.65% (-110.64% / 157.95%)
## 4:      -12.45% (-34.91% / 10%)     -22.56% (-50.62% / 5.5%)
## 5: -29.53% (-437.02% / 377.95%) -61.84% (-701.87% / 578.19%)
## 6:     +0.5% (-19.38% / 20.39%)   +85.22% (66.43% / 104.01%)
## 7:  +4.82% (-156.97% / 166.61%) +48.34% (-157.43% / 254.11%)
## 8:    -9.83% (-59.02% / 39.36%)    +28.41% (-36.7% / 93.53%)
## 9:    +63.43% (27.83% / 99.03%)    -0.63% (-47.22% / 45.95%)
##                            Myev                         Lano
## 1:      -1.02% (-6.02% / 3.98%)     -6.02% (-12.16% / 0.11%)
## 2:     +9.88% (-30.35% / 50.1%)    +2.29% (-57.63% / 62.21%)
## 3:  -33.34% (-126.69% / 60.01%)   +8.04% (-133.6% / 149.67%)
## 4:    -13.66% (-33.73% / 6.41%)    -7.95% (-36.15% / 20.25%)
## 5: -53.73% (-390.18% / 282.72%) -45.01% (-690.08% / 600.07%)
## 6:  +101.53% (84.69% / 118.37%) +135.27% (114.49% / 156.06%)
## 7:  +39.28% (-93.62% / 172.18%) +13.04% (-202.89% / 228.98%)
## 8:    +8.25% (-36.73% / 53.23%)    -17.24% (-83.68% / 49.2%)
## 9:   -19.97% (-54.43% / 14.48%)   +34.97% (-12.21% / 82.15%)
##                            Laci                         Epfu
## 1:      +1.59% (-4.59% / 7.77%)     -4.39% (-11.95% / 3.17%)
## 2:   -17.35% (-59.64% / 24.94%)   +19.08% (-34.58% / 72.74%)
## 3:  -12.11% (-111.07% / 86.84%) -29.44% (-176.37% / 117.49%)
## 4:    -9.54% (-29.96% / 10.88%)   -12.54% (-38.62% / 13.53%)
## 5: +146.15% (-198.61% / 490.9%) +39.45% (-482.12% / 561.02%)
## 6: +523.11% (498.79% / 547.43%)    +69.54% (43.76% / 95.32%)
## 7:  +29.22% (-111.16% / 169.6%) +50.02% (-158.81% / 258.85%)
## 8:   -19.74% (-65.73% / 26.24%)    -28.3% (-86.68% / 30.08%)
## 9:    +24.46% (-9.73% / 58.64%)   +82.29% (40.79% / 123.79%)
```

While we are quite familiar with what the predictor names represent at this point in the analysis, others may not be. Adding more descriptive labels to the table can be accomplished by adding a vector listing the more descriptive predictor names in the same order as the existing predictor names. After ensuring the short and long predictor names are properly aligned, we can assign the long names to the “Predictor” vector and delete the original vector of long names.

```
RealEfWide[,PredictorLong := c("Moon Illum. (%)", "Proportion Forested",
                               "Proportion Developed", "Water Distance (m)",
                               "Elevation (m)", "Ordinal Date (Days)",
                               "Brightness Index", "Proportion Cool Lights",
                               "Structure Index")]

RealEfWide
```

```
##      Predictor UnitChange                          Myyu
## 1:   MoonScale       0.20      +6.34% (-10.63% / 23.3%)
## 2: ForestScale       0.20     +0.65% (-71.18% / 72.49%)
## 3:  DevelScale       0.20  +15.09% (-178.78% / 208.96%)
## 4:  WaterScale     250.00    -22.99% (-63.25% / 17.27%)
## 5:   ElevScale      50.00 -52.19% (-1033.85% / 929.48%)
## 6:   YdayScale      30.00     +56.04% (1.51% / 110.58%)
## 7: BrightScale       5.00   -16.15% (-271.2% / 238.89%)
## 8:   CoolScale       0.20    +28.74% (-36.16% / 93.64%)
## 9:    StrScale       0.05     +3.24% (-54.63% / 61.12%)
##                            Myvo                         Mylu
## 1:      -2.09% (-8.29% / 4.11%)      +9.57% (4.58% / 14.56%)
## 2:    +89.11% (43.8% / 134.41%)   +57.22% (-1.45% / 115.89%)
## 3:   +4.78% (-111.13% / 120.7%) +23.65% (-110.64% / 157.95%)
## 4:      -12.45% (-34.91% / 10%)     -22.56% (-50.62% / 5.5%)
## 5: -29.53% (-437.02% / 377.95%) -61.84% (-701.87% / 578.19%)
## 6:     +0.5% (-19.38% / 20.39%)   +85.22% (66.43% / 104.01%)
## 7:  +4.82% (-156.97% / 166.61%) +48.34% (-157.43% / 254.11%)
## 8:    -9.83% (-59.02% / 39.36%)    +28.41% (-36.7% / 93.53%)
## 9:    +63.43% (27.83% / 99.03%)    -0.63% (-47.22% / 45.95%)
##                            Myev                         Lano
## 1:      -1.02% (-6.02% / 3.98%)     -6.02% (-12.16% / 0.11%)
## 2:     +9.88% (-30.35% / 50.1%)    +2.29% (-57.63% / 62.21%)
## 3:  -33.34% (-126.69% / 60.01%)   +8.04% (-133.6% / 149.67%)
## 4:    -13.66% (-33.73% / 6.41%)    -7.95% (-36.15% / 20.25%)
## 5: -53.73% (-390.18% / 282.72%) -45.01% (-690.08% / 600.07%)
## 6:  +101.53% (84.69% / 118.37%) +135.27% (114.49% / 156.06%)
## 7:  +39.28% (-93.62% / 172.18%) +13.04% (-202.89% / 228.98%)
## 8:    +8.25% (-36.73% / 53.23%)    -17.24% (-83.68% / 49.2%)
## 9:   -19.97% (-54.43% / 14.48%)   +34.97% (-12.21% / 82.15%)
##                            Laci                         Epfu
## 1:      +1.59% (-4.59% / 7.77%)     -4.39% (-11.95% / 3.17%)
## 2:   -17.35% (-59.64% / 24.94%)   +19.08% (-34.58% / 72.74%)
## 3:  -12.11% (-111.07% / 86.84%) -29.44% (-176.37% / 117.49%)
## 4:    -9.54% (-29.96% / 10.88%)   -12.54% (-38.62% / 13.53%)
## 5: +146.15% (-198.61% / 490.9%) +39.45% (-482.12% / 561.02%)
## 6: +523.11% (498.79% / 547.43%)    +69.54% (43.76% / 95.32%)
## 7:  +29.22% (-111.16% / 169.6%) +50.02% (-158.81% / 258.85%)
## 8:   -19.74% (-65.73% / 26.24%)    -28.3% (-86.68% / 30.08%)
## 9:    +24.46% (-9.73% / 58.64%)   +82.29% (40.79% / 123.79%)
##             PredictorLong
## 1:        Moon Illum. (%)
## 2:    Proportion Forested
## 3:   Proportion Developed
## 4:     Water Distance (m)
## 5:          Elevation (m)
## 6:    Ordinal Date (Days)
## 7:       Brightness Index
## 8: Proportion Cool Lights
## 9:        Structure Index
```

```
RealEfWide[,Predictor := PredictorLong]
RealEfWide[,PredictorLong := NULL]

RealEfWide
```

```
##                 Predictor UnitChange                          Myyu
## 1:        Moon Illum. (%)       0.20      +6.34% (-10.63% / 23.3%)
## 2:    Proportion Forested       0.20     +0.65% (-71.18% / 72.49%)
## 3:   Proportion Developed       0.20  +15.09% (-178.78% / 208.96%)
## 4:     Water Distance (m)     250.00    -22.99% (-63.25% / 17.27%)
## 5:          Elevation (m)      50.00 -52.19% (-1033.85% / 929.48%)
## 6:    Ordinal Date (Days)      30.00     +56.04% (1.51% / 110.58%)
## 7:       Brightness Index       5.00   -16.15% (-271.2% / 238.89%)
## 8: Proportion Cool Lights       0.20    +28.74% (-36.16% / 93.64%)
## 9:        Structure Index       0.05     +3.24% (-54.63% / 61.12%)
##                            Myvo                         Mylu
## 1:      -2.09% (-8.29% / 4.11%)      +9.57% (4.58% / 14.56%)
## 2:    +89.11% (43.8% / 134.41%)   +57.22% (-1.45% / 115.89%)
## 3:   +4.78% (-111.13% / 120.7%) +23.65% (-110.64% / 157.95%)
## 4:      -12.45% (-34.91% / 10%)     -22.56% (-50.62% / 5.5%)
## 5: -29.53% (-437.02% / 377.95%) -61.84% (-701.87% / 578.19%)
## 6:     +0.5% (-19.38% / 20.39%)   +85.22% (66.43% / 104.01%)
## 7:  +4.82% (-156.97% / 166.61%) +48.34% (-157.43% / 254.11%)
## 8:    -9.83% (-59.02% / 39.36%)    +28.41% (-36.7% / 93.53%)
## 9:    +63.43% (27.83% / 99.03%)    -0.63% (-47.22% / 45.95%)
##                            Myev                         Lano
## 1:      -1.02% (-6.02% / 3.98%)     -6.02% (-12.16% / 0.11%)
## 2:     +9.88% (-30.35% / 50.1%)    +2.29% (-57.63% / 62.21%)
## 3:  -33.34% (-126.69% / 60.01%)   +8.04% (-133.6% / 149.67%)
## 4:    -13.66% (-33.73% / 6.41%)    -7.95% (-36.15% / 20.25%)
## 5: -53.73% (-390.18% / 282.72%) -45.01% (-690.08% / 600.07%)
## 6:  +101.53% (84.69% / 118.37%) +135.27% (114.49% / 156.06%)
## 7:  +39.28% (-93.62% / 172.18%) +13.04% (-202.89% / 228.98%)
## 8:    +8.25% (-36.73% / 53.23%)    -17.24% (-83.68% / 49.2%)
## 9:   -19.97% (-54.43% / 14.48%)   +34.97% (-12.21% / 82.15%)
##                            Laci                         Epfu
## 1:      +1.59% (-4.59% / 7.77%)     -4.39% (-11.95% / 3.17%)
## 2:   -17.35% (-59.64% / 24.94%)   +19.08% (-34.58% / 72.74%)
## 3:  -12.11% (-111.07% / 86.84%) -29.44% (-176.37% / 117.49%)
## 4:    -9.54% (-29.96% / 10.88%)   -12.54% (-38.62% / 13.53%)
## 5: +146.15% (-198.61% / 490.9%) +39.45% (-482.12% / 561.02%)
## 6: +523.11% (498.79% / 547.43%)    +69.54% (43.76% / 95.32%)
## 7:  +29.22% (-111.16% / 169.6%) +50.02% (-158.81% / 258.85%)
## 8:   -19.74% (-65.73% / 26.24%)    -28.3% (-86.68% / 30.08%)
## 9:    +24.46% (-9.73% / 58.64%)   +82.29% (40.79% / 123.79%)
```

We can take a similar approach to further increase the interpretability of the table by adding a vector that describes whether predictors are classified as anthropogenic or natural, and grouping predictors with a common classification in our results table. Just as we did above, we will need to detail the classification for each predictor in the same order that the predictors are listed.

```
# Creates a vector of predictor classifications in the same order that the predictors are listed
RealEfWide[,PredNature := c("Natural", "Natural", "Anthropogenic", "Natural",
                            "Natural", "Natural", "Anthropogenic", "Anthropogenic",
                            "Anthropogenic")]

# Groups predictors with a common classification together in the results table
setorder(RealEfWide, PredNature)

RealEfWide
```

```
##                 Predictor UnitChange                          Myyu
## 1:   Proportion Developed       0.20  +15.09% (-178.78% / 208.96%)
## 2:       Brightness Index       5.00   -16.15% (-271.2% / 238.89%)
## 3: Proportion Cool Lights       0.20    +28.74% (-36.16% / 93.64%)
## 4:        Structure Index       0.05     +3.24% (-54.63% / 61.12%)
## 5:        Moon Illum. (%)       0.20      +6.34% (-10.63% / 23.3%)
## 6:    Proportion Forested       0.20     +0.65% (-71.18% / 72.49%)
## 7:     Water Distance (m)     250.00    -22.99% (-63.25% / 17.27%)
## 8:          Elevation (m)      50.00 -52.19% (-1033.85% / 929.48%)
## 9:    Ordinal Date (Days)      30.00     +56.04% (1.51% / 110.58%)
##                            Myvo                         Mylu
## 1:   +4.78% (-111.13% / 120.7%) +23.65% (-110.64% / 157.95%)
## 2:  +4.82% (-156.97% / 166.61%) +48.34% (-157.43% / 254.11%)
## 3:    -9.83% (-59.02% / 39.36%)    +28.41% (-36.7% / 93.53%)
## 4:    +63.43% (27.83% / 99.03%)    -0.63% (-47.22% / 45.95%)
## 5:      -2.09% (-8.29% / 4.11%)      +9.57% (4.58% / 14.56%)
## 6:    +89.11% (43.8% / 134.41%)   +57.22% (-1.45% / 115.89%)
## 7:      -12.45% (-34.91% / 10%)     -22.56% (-50.62% / 5.5%)
## 8: -29.53% (-437.02% / 377.95%) -61.84% (-701.87% / 578.19%)
## 9:     +0.5% (-19.38% / 20.39%)   +85.22% (66.43% / 104.01%)
##                            Myev                         Lano
## 1:  -33.34% (-126.69% / 60.01%)   +8.04% (-133.6% / 149.67%)
## 2:  +39.28% (-93.62% / 172.18%) +13.04% (-202.89% / 228.98%)
## 3:    +8.25% (-36.73% / 53.23%)    -17.24% (-83.68% / 49.2%)
## 4:   -19.97% (-54.43% / 14.48%)   +34.97% (-12.21% / 82.15%)
## 5:      -1.02% (-6.02% / 3.98%)     -6.02% (-12.16% / 0.11%)
## 6:     +9.88% (-30.35% / 50.1%)    +2.29% (-57.63% / 62.21%)
## 7:    -13.66% (-33.73% / 6.41%)    -7.95% (-36.15% / 20.25%)
## 8: -53.73% (-390.18% / 282.72%) -45.01% (-690.08% / 600.07%)
## 9:  +101.53% (84.69% / 118.37%) +135.27% (114.49% / 156.06%)
##                            Laci                         Epfu    PredNature
## 1:  -12.11% (-111.07% / 86.84%) -29.44% (-176.37% / 117.49%) Anthropogenic
## 2:  +29.22% (-111.16% / 169.6%) +50.02% (-158.81% / 258.85%) Anthropogenic
## 3:   -19.74% (-65.73% / 26.24%)    -28.3% (-86.68% / 30.08%) Anthropogenic
## 4:    +24.46% (-9.73% / 58.64%)   +82.29% (40.79% / 123.79%) Anthropogenic
## 5:      +1.59% (-4.59% / 7.77%)     -4.39% (-11.95% / 3.17%)       Natural
## 6:   -17.35% (-59.64% / 24.94%)   +19.08% (-34.58% / 72.74%)       Natural
## 7:    -9.54% (-29.96% / 10.88%)   -12.54% (-38.62% / 13.53%)       Natural
## 8: +146.15% (-198.61% / 490.9%) +39.45% (-482.12% / 561.02%)       Natural
## 9: +523.11% (498.79% / 547.43%)    +69.54% (43.76% / 95.32%)       Natural
```

Our table is coming along well, but the species are in reverse alphabetical order. If we want the species in alphabetical order, we will have to specify the column order manually using `setcolorder()`.

```
setcolorder(x = RealEfWide, neworder = c("PredNature", "Predictor", "UnitChange",
                                         "Epfu", "Laci", "Lano", 
                                         "Myev", "Mylu", "Myvo",
                                         "Myyu"))
```

At this point we have a relatively readable results table, but it isn’t aesthetically pleasing and we cannot use it in another document without exporting it as a delimited file and altering it in a spreadsheet program. Instead of taking that route, we can use the `kableExtra` package (installed with `EcoCountHelper`) to produce an image of the table that is attractive and portable.

To begin this process, we should change the names of any vectors that are cryptic. In this case, we should change the “UnitChange” vector name to something more descriptive and intuitive such as “Unit Increase.”

```
setnames(RealEfWide, old = "UnitChange",
         new = "Unit Increase",
         skip_absent = T)
```

We now have a table that looks like the one below.

```
RealEfWide
```

```
##       PredNature              Predictor Unit Increase
## 1: Anthropogenic   Proportion Developed          0.20
## 2: Anthropogenic       Brightness Index          5.00
## 3: Anthropogenic Proportion Cool Lights          0.20
## 4: Anthropogenic        Structure Index          0.05
## 5:       Natural        Moon Illum. (%)          0.20
## 6:       Natural    Proportion Forested          0.20
## 7:       Natural     Water Distance (m)        250.00
## 8:       Natural          Elevation (m)         50.00
## 9:       Natural    Ordinal Date (Days)         30.00
##                            Epfu                         Laci
## 1: -29.44% (-176.37% / 117.49%)  -12.11% (-111.07% / 86.84%)
## 2: +50.02% (-158.81% / 258.85%)  +29.22% (-111.16% / 169.6%)
## 3:    -28.3% (-86.68% / 30.08%)   -19.74% (-65.73% / 26.24%)
## 4:   +82.29% (40.79% / 123.79%)    +24.46% (-9.73% / 58.64%)
## 5:     -4.39% (-11.95% / 3.17%)      +1.59% (-4.59% / 7.77%)
## 6:   +19.08% (-34.58% / 72.74%)   -17.35% (-59.64% / 24.94%)
## 7:   -12.54% (-38.62% / 13.53%)    -9.54% (-29.96% / 10.88%)
## 8: +39.45% (-482.12% / 561.02%) +146.15% (-198.61% / 490.9%)
## 9:    +69.54% (43.76% / 95.32%) +523.11% (498.79% / 547.43%)
##                            Lano                         Myev
## 1:   +8.04% (-133.6% / 149.67%)  -33.34% (-126.69% / 60.01%)
## 2: +13.04% (-202.89% / 228.98%)  +39.28% (-93.62% / 172.18%)
## 3:    -17.24% (-83.68% / 49.2%)    +8.25% (-36.73% / 53.23%)
## 4:   +34.97% (-12.21% / 82.15%)   -19.97% (-54.43% / 14.48%)
## 5:     -6.02% (-12.16% / 0.11%)      -1.02% (-6.02% / 3.98%)
## 6:    +2.29% (-57.63% / 62.21%)     +9.88% (-30.35% / 50.1%)
## 7:    -7.95% (-36.15% / 20.25%)    -13.66% (-33.73% / 6.41%)
## 8: -45.01% (-690.08% / 600.07%) -53.73% (-390.18% / 282.72%)
## 9: +135.27% (114.49% / 156.06%)  +101.53% (84.69% / 118.37%)
##                            Mylu                         Myvo
## 1: +23.65% (-110.64% / 157.95%)   +4.78% (-111.13% / 120.7%)
## 2: +48.34% (-157.43% / 254.11%)  +4.82% (-156.97% / 166.61%)
## 3:    +28.41% (-36.7% / 93.53%)    -9.83% (-59.02% / 39.36%)
## 4:    -0.63% (-47.22% / 45.95%)    +63.43% (27.83% / 99.03%)
## 5:      +9.57% (4.58% / 14.56%)      -2.09% (-8.29% / 4.11%)
## 6:   +57.22% (-1.45% / 115.89%)    +89.11% (43.8% / 134.41%)
## 7:     -22.56% (-50.62% / 5.5%)      -12.45% (-34.91% / 10%)
## 8: -61.84% (-701.87% / 578.19%) -29.53% (-437.02% / 377.95%)
## 9:   +85.22% (66.43% / 104.01%)     +0.5% (-19.38% / 20.39%)
##                             Myyu
## 1:  +15.09% (-178.78% / 208.96%)
## 2:   -16.15% (-271.2% / 238.89%)
## 3:    +28.74% (-36.16% / 93.64%)
## 4:     +3.24% (-54.63% / 61.12%)
## 5:      +6.34% (-10.63% / 23.3%)
## 6:     +0.65% (-71.18% / 72.49%)
## 7:    -22.99% (-63.25% / 17.27%)
## 8: -52.19% (-1033.85% / 929.48%)
## 9:     +56.04% (1.51% / 110.58%)
```

If you would like to modify the table above in a spreadsheet program or paste the table into a document for further formatting, writing the table to a file is the next step. You can use base R’s `write.csv()` function for writing tables to external files as is shown below.

```
write.csv(RealEfWide, SaveDir, row.names = F)
```

If you plan to format the table in R and export the formatted table as an image, there is one final step we can execute. Rather than having “Anthropogenic” and “Natural” shown in each row of our formatted table, we can manually specify rows to be grouped as well as the value that should be shown for each group. Before creating a formatted table, we can record the rows that fall under each predictor classification (anthropogenic or natural) and delete the “PredNature” vector. From the table above, we know that anthropogenic predictors occupy rows one through four, and natural predictors occupy rows five through nine. With that information recorded, we can remove the “PredNature” vector from the “RealEfWide” table.

```
RealEfWide[,PredNature := NULL]
```

Now we can generate a formatted table using `kableExtra` using the code below. The “%>%” operator is used for piping, which is the process of using the object generated by a line or chunk of code in the following line or chunk without having to permanently assign the intermediate object to the global environment.

```
library(kableExtra)

# Creates table and center aligns all cell values
RealEfKable <- kable(RealEfWide, align = "c") %>%
  # Adds a grey stripe on every other line
  row_spec(seq(2, nrow(RealEfWide), 2), background = "grey") %>%
  # makes text color in all rows black
  row_spec(1:nrow(RealEfWide), color = "black") %>%
  # Adds "Anthropogenic" group indicator for rows 1 to 4
  group_rows("Anthropogenic", 1, 4) %>%
  # Adds "Natural" group indicator for rows 5 to 9
  group_rows("Natural", 5, 9)

RealEfKable
```

| Predictor | Unit Increase | Epfu | Laci | Lano | Myev | Mylu | Myvo | Myyu |
| --- | --- | --- | --- | --- | --- | --- | --- | --- |
| **Anthropogenic** | | | | | | | | |
| Proportion Developed | 0.20 | -29.44% (-176.37% / 117.49%) | -12.11% (-111.07% / 86.84%) | +8.04% (-133.6% / 149.67%) | -33.34% (-126.69% / 60.01%) | +23.65% (-110.64% / 157.95%) | +4.78% (-111.13% / 120.7%) | +15.09% (-178.78% / 208.96%) |
| Brightness Index | 5.00 | +50.02% (-158.81% / 258.85%) | +29.22% (-111.16% / 169.6%) | +13.04% (-202.89% / 228.98%) | +39.28% (-93.62% / 172.18%) | +48.34% (-157.43% / 254.11%) | +4.82% (-156.97% / 166.61%) | -16.15% (-271.2% / 238.89%) |
| Proportion Cool Lights | 0.20 | -28.3% (-86.68% / 30.08%) | -19.74% (-65.73% / 26.24%) | -17.24% (-83.68% / 49.2%) | +8.25% (-36.73% / 53.23%) | +28.41% (-36.7% / 93.53%) | -9.83% (-59.02% / 39.36%) | +28.74% (-36.16% / 93.64%) |
| Structure Index | 0.05 | +82.29% (40.79% / 123.79%) | +24.46% (-9.73% / 58.64%) | +34.97% (-12.21% / 82.15%) | -19.97% (-54.43% / 14.48%) | -0.63% (-47.22% / 45.95%) | +63.43% (27.83% / 99.03%) | +3.24% (-54.63% / 61.12%) |
| **Natural** | | | | | | | | |
| Moon Illum. (%) | 0.20 | -4.39% (-11.95% / 3.17%) | +1.59% (-4.59% / 7.77%) | -6.02% (-12.16% / 0.11%) | -1.02% (-6.02% / 3.98%) | +9.57% (4.58% / 14.56%) | -2.09% (-8.29% / 4.11%) | +6.34% (-10.63% / 23.3%) |
| Proportion Forested | 0.20 | +19.08% (-34.58% / 72.74%) | -17.35% (-59.64% / 24.94%) | +2.29% (-57.63% / 62.21%) | +9.88% (-30.35% / 50.1%) | +57.22% (-1.45% / 115.89%) | +89.11% (43.8% / 134.41%) | +0.65% (-71.18% / 72.49%) |
| Water Distance (m) | 250.00 | -12.54% (-38.62% / 13.53%) | -9.54% (-29.96% / 10.88%) | -7.95% (-36.15% / 20.25%) | -13.66% (-33.73% / 6.41%) | -22.56% (-50.62% / 5.5%) | -12.45% (-34.91% / 10%) | -22.99% (-63.25% / 17.27%) |
| Elevation (m) | 50.00 | +39.45% (-482.12% / 561.02%) | +146.15% (-198.61% / 490.9%) | -45.01% (-690.08% / 600.07%) | -53.73% (-390.18% / 282.72%) | -61.84% (-701.87% / 578.19%) | -29.53% (-437.02% / 377.95%) | -52.19% (-1033.85% / 929.48%) |
| Ordinal Date (Days) | 30.00 | +69.54% (43.76% / 95.32%) | +523.11% (498.79% / 547.43%) | +135.27% (114.49% / 156.06%) | +101.53% (84.69% / 118.37%) | +85.22% (66.43% / 104.01%) | +0.5% (-19.38% / 20.39%) | +56.04% (1.51% / 110.58%) |

The tables generated by `kableExtra` can be exported manually by using the “Export” GUI in the viewer window, or can be saved to a file using using the `save_kable()` function. For more information on formatting kableExtra tables, see this kableExtra vignette.

## Long-form Analysis

Analyzing long-form data with `EcoCountHelper` is a nearly identical process to analyzing wide-form data with `EcoCountHelper`. Most of the changes to the process involve the addition of one or two arguments in the long-form versions (ending with “Long” instead of “Wide”) of certain functions. The two most common additional arguments in the long-form functions are “GroupCol” and “CountCol.” Because long-form data does not have separate columns for count data belonging to each response group, there is a single column indicating response group membership (in this example, species) and another column indicating the count value. “GroupCol” and “CountCol” arguments require a character value specifying the name of the vector containing data specifying group membership and count value, respectively.

You will also encounter a logical “Looped” argument in the `ResidPlotLong()` function. If modeling long-form data with a single model for each response group (e.g., one GLMM with a Poisson error distribution and no zero-inflated model), you may loop through your data by using code akin to the code chunk below:

```
for(i in unique(BatDataLong$Species)){
  TmpData <- BatDataLong[Species == i,]
  
  SpPoi <- tryCatch({glmmTMB(Count ~ StrScale + CoolScale + BrightScale + Year +
                               YdayScale + ElevScale + WaterScale + DevelScale + ForestScale +
                               MoonScale + (1|Site),
                   data = TmpData, family = poisson(link = "log"))},
                   error = function(cond){return(NA)},
                   warning = function(cond){return(NA)})
  
  assign(paste0(i, "_Poi"), SpPoi, pos = .GlobalEnv)
}
```

Because the “data” argument is supplied “TmpData” which is an ephemeral object, the residual simulation process that `ResidPlotLong()` borrows from the `DHARMa` package searches for “TmpData” but cannot find it, and thus returns an error. By supplying `TRUE` to the “Looped” argument, `ResidPlotLong()` assumes that the the “data” value in each model needs to be updated to point to the permanent data set indicated by `ResidPlotLong()`’s “Data” argument (in this case, “BatDataLong”) rather than an ephemeral object.

Finally, there is a “GroupPat” argument in `RealEffectTabLong()` that must be supplied a regular expression to identify the response group to which each model supplied to the “Models” argument belongs. The regular expression should be the same as the values supplied to the “GroupPat” argument in all other functions. If model names start with the response group abbreviation followed by an underscore as was suggested earlier in this document, the default value of “^[[:alnum:]]+” will be sufficient.

## References

Bolker B. 2016. Getting started with the glmmTMB package. *Vienna, Austria: R Foundation for Statistical Computing. software*.

Bolker BM, Brooks ME, Clark CJ, Geange SW, Poulsen JR, Stevens MHH, White J-SS. 2009. Generalized linear mixed models: A practical guide for ecology and evolution. *Trends in ecology & evolution* 24:127–135. DOI: 10.1016/j.tree.2008.10.008.

Brooks ME, Kristensen K, Benthem KJ van, Magnusson A, Berg CW, Nielsen A, Skaug HJ, Machler M, Bolker BM. 2017a. glmmTMB balances speed and flexibility among packages for zero-inflated generalized linear mixed modeling. *The R journal* 9:378–400. DOI: 10.3929/ethz-b-000240890.

Brooks ME, Kristensen K, Benthem KJ van, Magnusson A, Berg CW, Nielsen A, Skaug HJ, Mächler M, Bolker BM. 2017b. Modeling zero-inflated count data with glmmTMB. *BioRxiv*:132753. DOI: 10.1101/132753.

Gelman A. 2008. Scaling regression inputs by dividing by two standard deviations. *Statistics in Medicine* 27:2865–2873. DOI: 10.1002/sim.3107.

Gelman A, Hill J. 2006. *Data analysis using regression and multilevel/hierarchical models*. Cambridge university press.

Harrison XA, Donaldson L, Correa-Cano ME, Evans J, Fisher DN, Goodwin CED, Robinson BS, Hodgson DJ, Inger R. 2018. A brief introduction to mixed effects modelling and multi-model inference in ecology. *PeerJ* 6. DOI: 10.7717/peerj.4794.

Martin TG, Wintle BA, Rhodes JR, Kuhnert PM, Field SA, Low-Choy SJ, Tyre AJ, Possingham HP. 2005. Zero tolerance ecology: Improving ecological inference by modelling the source of zero observations. *Ecology letters* 8:1235–1246.
